# MicroRNA-124: A Key Player in Microglia-Mediated Inflammation in Neurological Diseases

**DOI:** 10.3389/fncel.2021.771898

**Published:** 2021-11-02

**Authors:** Jiuhan Zhao, Zhenwei He, Jialu Wang

**Affiliations:** ^1^Department of Neurology, First Affiliated Hospital of China Medical University, Shenyang, China; ^2^Department of Neurology, The Fourth Affiliated Hospital of China Medical University, Shenyang, China

**Keywords:** microRNA-124, neurological disorders, microglia, biomarker, therapeutic target

## Abstract

Neurological disorders are mainly characterized by progressive neuron loss and neurological deterioration, which cause human disability and death. However, many types of neurological disorders have similar pathological mechanisms, including the neuroinflammatory response. Various microRNAs (miRs), such as miR-21, miR-124, miR-146a, and miR-132 were recently shown to affect a broad spectrum of biological functions in the central nervous system (CNS). Microglia are innate immune cells with important roles in the physiological and pathological activities of the CNS. Recently, abnormal expression of miR-124 was shown to be associated with the occurrence and development of various diseases in CNS via regulating microglia function. In addition, miR-124 is a promising biomarker and therapeutic target. Studies on the role of miR-124 in regulating microglia function involved in pathogenesis of neurological disorders at different stages will provide new ideas for the use of miR-124 as a therapeutic target for different CNS diseases.

## Introduction

Progressive loss of neurons and deterioration of neurological symptoms are the pathological basis and main features of neurological disorders. Neurological disorders have diverse etiologies and progress rapidly, which can be disabling and fatal (Ayaz et al., 2019). The main causes of neurological disorders include cerebrovascular diseases (CVDs), neurodegenerative diseases (NDDs), Alzheimer’s disease (AD), Parkinson’s disease (PD), motor neuron disease, Huntington’s disease (HD), epileptic diseases, neurological tumors, traumatic diseases, and neuro autoimmune diseases (Santalucia, 2008; Song and Suk, 2017). Although the complex mechanisms of various neurological disorders have been widely studied, their precise etiologies remain largely unknown. However, most neurological disorders share common pathophysiological mechanisms and disease characteristics, particularly activated microglia (MG)-mediated neuroinflammatory responses.

MicroRNA (miR) is a multifunctional endogenous non-coding small molecule RNA, consisting of 18–25 nucleotide sequences and an incomplete 3′-untranslated region of the target mRNA molecule complementary combination. The miRs regulate gene expression and protein synthesis at the post-transcriptional level and participate in cell proliferation, differentiation, apoptosis, and other life activities (Yao et al., 2020). Emerging studies have shown that various miRs, such as miR-21, miR-124, miR-146a, and miR-132, play key roles in neurological disorders and may be feasible therapeutic targets (Gascon et al., 2014; Devaux et al., 2016). The miR-124 is among the most abundant miRs in the mammalian nervous system, showing a much higher level in the central nervous system (CNS) than in other organs and accounting for 5–48% of the total miRNA content in the cerebral cortex (Landgraf et al., 2007; Treiber et al., 2012). miR-124 has three immature precursor sequences, miR-124-1, miR-124-2, and miR-124-3, which are on chromosomes 8p23.1, 8q12.3, and 20q13.33, respectively (Lagos-Quintana et al., 2002). miR-124 expression first occurs during neural differentiation and reaches a peak in mature neurons (Sonntag et al., 2012). During CNS development and mature nerve formation, miR-124 can inhibit cell proliferation and promote cell differentiation, thereby regulating nerve differentiation (Makeyev et al., 2007).

Microglia are innate immune cells of the CNS and play important roles as “executors” in neuroinflammation, functioning in immune monitoring and defense (Aguzzi et al., 2013). Previous studies suggested that miR-124 can regulate the polarization state of MG and that up-regulating the expression level of miR-124 can promote the transformation of MG from the pro-inflammatory M1 type to the anti-inflammatory M2 type (Yu et al., 2017; Periyasamy et al., 2018). miR-124 expression in MG has also been shown to reduce inflammation by downregulating tumor necrosis factor α (TNF-α) and major histocompatibility complex II, as well as by reducing reactive oxygen species (Louw et al., 2016). In addition, miR-124 acts as a key regulator of MG quiescence in the CNS and as a previously unknown modulator of monocyte and macrophage activation (Ponomarev et al., 2011). Herein, we review the role of miR-124 in regulating MG function in the pathogenesis of different neurological disorders, its target genes, changes in expression levels, and the pathogenic pathways that may be involved. We also describe the potential of miR-124 as a biomarker and therapeutic target (Table 1) for the diagnosis and prognosis of neurological disorders.

**TABLE 1:** Circulating miR-124 expression as circulating biomarker in neurological disorders.

**Neurological disorders**	**Disease model**	**Sample**	**miRNA expression change**	**Target genes/related pathway/function**	**References**
Acute ischemic stroke, AIS	Patient	Plasma	miR-124-3p, increased	AIS diagnosis	Rainer et al., 2016
Acute ischemic stroke, AIS	Patient	Plasma	miR-124-3p, increased	AIS patient severity and prognosis	He et al., 2019
Acute ischemic stroke, AIS	Patient	Plasma	miR-124, decreased within 24 h	AIS diagnosis	Sun et al., 2019
Acute ischemic stroke, AIS	Patient	Serum	miR-124, decreased within 24 h	AIS diagnosis, predicting infarction volume	Liu et al., 2015
Intracerebral hemorrhage, ICH	Patient	Plasma	miR-124, increased within 24 h	ICH diagnosis	Wang Z. et al., 2018
Stroke	Patient	Plasma	miR-124-3p, increased	ICH and AIS discrimination	Leung et al., 2014
Parkinson’s disease, PD	Patient	Plasma	miR-124, decreased	PD diagnosis	Li et al., 2017
Parkinson’s disease, PD	Patient	Plasma	miR-124-3p, decreased	PD diagnosis	Ravanidis et al., 2020
Multiple sclerosis, MS	Patient	Monocytes	miR-124, decreased	Progressive MS diagnosis	Amoruso et al., 2020
Traumatic brain injury, TBI	Patient	Plasma	miR-124-3p, increased	TBI diagnosis	Vuokila et al., 2020
Traumatic brain injury, TBI	Patient	Plasma	miR-124-3p, increased	Evaluating TBI severity	Schindler et al., 2020
Traumatic brain injury, TBI	Patient	Serum	miR-124-3p, increased	TBI diagnosis	O’connell et al., 2020

## MicroRNA-124 Roles in Neurological Disorders

**FIGURE 1:**
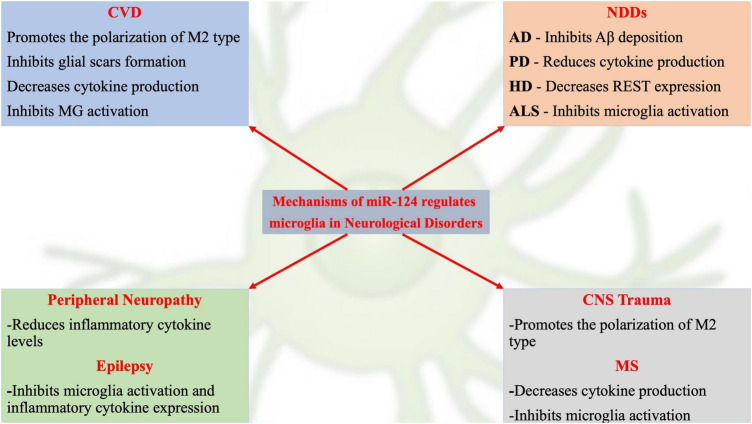
Schematic representation of current hot topics and emerging mechanisms of miR-124 in regulating microglia in neurological disorders.

Although recent studies have revealed initial information on miR-124-related signaling pathway mechanisms, many potential mechanisms remain to be identified. Dysregulated miR-124 expression may be involved in the occurrence and development of various neurological disorders by regulating MG function (Figure 1). For example, decreased miR-124 expression in the nervous system is often an important link in neurodegenerative and glioma diseases (Kanagaraj et al., 2014; Wu et al., 2018; Serpe et al., 2021). A recent review on the role of miR-124 in the pathogenesis of neurological disorders described the pathophysiological processes of neurological disorders and novel therapeutic strategies for treating neurological disorders (Han et al., 2019).

## MicroRNA-124 and Cerebrovascular Diseases

Cerebrovascular diseases can lead to death or long-term disability and is a worldwide health concern. Plasma miR-124-3p levels were found to be elevated in patients with acute ischemic stroke (AIS) (Badacz et al., 2021), and the miR-124-3p level was positively correlated with the poor prognosis of these patients based on the modified Rankin score (Rainer et al., 2016). Higher plasma miR-124-3p levels were suggested to be associated with unfavorable outcomes in patients with AIS undergoing thrombolysis, and miR-124-3p is closely related with the severity of stroke (He et al., 2019). miR-124 levels dynamically changed in patients with AIS (Ji et al., 2016), rapidly decreasing within 24 h and gradually increasing at 48 h after ischemia (Sun et al., 2019). However, the opposite conclusion was reported in several other studies. The serum miR-124 level was decreased within 24 h after stroke onset and was negatively correlated with high-sensitivity C-reactive protein levels and the infarct volume; the authors hypothesized that serum miR-124 was suppressed in AIS, thus facilitating neuroinflammation and brain injury (Liu et al., 2015). Intracerebral hemorrhage (ICH) accounts for 15% of stroke cases, showing a mortality rate as high as 50% within the first month of ICH onset, with half of these deaths occurring within 48 h. Thus, rapid diagnosis and timely treatment are important for patients with ICH (Broderick, 1993; Burke and Venketasubramanian, 2006). In a previous study, the average plasma miR-124 concentrations increased by more than 100-fold in 24 h, and subsequently decreased on days 2, 7, and 14, reaching nearly normal levels on day 30 (Wang Z. et al., 2018). miR-124-3p levels were investigated as diagnostic biomarkers of acute stroke; plasma miR-124-3p levels were higher in patients with ICH than in those with AIS within 24 h of symptom onset. Therefore, the miR-124-3p level was suggested as a diagnostic biomarker for discriminating ICH from AIS (Leung et al., 2014). In other studies, miR-124 decreased immediately after carotid arterial injury but dramatically increased at days 7 and 14 after injury. Thus, miR-124 was proposed as a novel regulator of vascular smooth muscle cell proliferation and involved in the development of neointimal proliferation (Choe et al., 2017).

Additional studies in animal models have been performed to determine the role of miR-124 in the pathophysiological mechanism of CVD (Volný et al., 2015). The miR-124 expression level was significantly decreased in cerebral infarction mice group compared to that in the sham group; miR-124 exerted neuroprotective and anti-inflammatory effects in mice with cerebral infarction by shifting the polarization of MG/macrophages into the beneficial, anti-inflammatory M2 type MG (Hamzei Taj et al., 2016). Early miR-124 injection significantly increases neuronal survival and the number of M2-like polarized MG. Furthermore, the stroke lesion core was significantly reduced over time following early miR-124 injection. These neuroprotective and anti-inflammatory effects of early miR-124 treatment were pronounced during the first week with Arg-1 (a marker for M2 phenotype), indicating miR-124 as a novel therapeutic agent for neuroprotection after stroke (Hamzei Taj et al., 2016).

Glial scars present a major obstacle to neuronal regeneration after ischemic stroke. Knockdown of miR-124 in M2 type MG small extracellular vesicles inhibited glial scar formation and promoted stroke recovery (Li et al., 2021). MG contributed to the inflammatory response and exacerbated ICH-induced secondary injury in mice. miR-124 overexpression decreased pro-inflammatory cytokine levels, indicating that this miRNA ameliorates ICH-induced inflammatory injury by modulating MG polarization toward the M2 phenotype (Yu et al., 2017). In an experimental rat subarachnoid hemorrhage model, Chen et al. demonstrated that delivery of exosomal miR-124 from neurons to MG was significantly reduced, accompanied by increased C/EBPα expression, which was inhibited by CX3CL1/CX3CR1 overexpression; thus, the CX3CL1/CX3CR1 axis may exert protective roles in subarachnoid hemorrhage by promoting the delivery of exosomal miR-124 to MG and inhibiting MG activation as well as the neuroinflammatory response (Chen et al., 2020).

Based on the above reports, although the circulating miR-124 expression level was not consistent among different CVD studies, in patients with AIS, the miR-124 level was elevated in most studies and associated with the poor prognosis of patients. miR-124 upregulation improved the prognosis of patients with CVD and reduced nerve cell injury through various mechanisms, such as the anti-inflammatory response, thereby playing a role in repairing the damaged CNS. However, the role of miR-124 as a protective agent for CVD by regulating miR-124 expression levels and the most effective method for miR-124 application requires further exploration.

## MicroRNA-124 and Neurodegenerative Diseases

### Alzheimer’s Disease

Alzheimer’s disease is a progressive NDD with an insidious onset. Clinically, AD is characterized by general dementia such as memory impairment, aphasia, apraxia, agnosia, visual space impairment, executive dysfunction, and personality and behavioral changes (Uddin et al., 2018). miR-124 is involved in the pathogenesis of AD through various mechanisms including MG activation and neuroinflammation (Bahlakeh et al., 2021).

Beta-site amyloid precursor protein cleaving enzyme 1 (BACE1) is a critical enzyme that regulates the production of amyloid-β (Aβ), which is abnormally upregulated in AD (Peng et al., 2017). Upregulated MG exosomes miR-124-3p (exo-miR-124-3p) alleviated neurodegeneration in repetitive scratch-injured neurons *in vitro*. Furthermore, miR-124-3p targets Rela (an inhibitory transcription factor of ApoE), which may promote Aβ proteolytic breakdown and inhibit Aβ abnormalities (Ge et al., 2020). miR-124 is the critical regulatory factor in the hypoxia/Aβ-miR-124-BACE1-Aβ cycle. Additionally, activation of the EPAC-Rap1 pathway was shown to be involved in inhibiting miR-124 in the hippocampus under hypoxia or Aβ insult (Zhang et al., 2017). miR-124 expression was downregulated with aging and showed a decreased ability to regulate ApoE-dependent Aβ uptake by targeting regulatory factor X1 transcripts on BV2 MG.

Tau hyperphosphorylation forms neurofibrillary tangles, a crucial event in the pathogenesis of AD. miR-124-3p was shown to inhibit abnormal tau hyperphosphorylation by regulating the caveolin-1-PI3K/Akt/GSK3β pathway in AD (Kang et al., 2017). Abnormal activation of cyclin-dependent kinase-5 (CDK5) is involved in controlling hyperphosphorylation, which is mediated by calpain-induced cleavage of p35 to p25. miR-124-3p is a calpain-targeting miR which may inhibit the protein translation of calpain. An inverse correlation was found between miR-124-3p and calpain levels in AD specimens. Furthermore, miR-124-3p overexpression significantly reduced Aβ deposition and improved AD mouse behavior through posttranscriptional control of calpain (Zhou et al., 2019).

Synaptic loss is an early pathological event in AD. Although the underlying molecular mechanism is unclear, miRNAs have been suggested as important regulators of synaptic function and memory (Selkoe, 2002). Protein tyrosine phosphatase non-receptor type 1 (PTPN1) reduction dramatically induces synaptic impairments and memory decline. Wang et al. demonstrated the PTPN1 is a direct target of miR-124, and the miR-124/PTPN1 pathway is involved in synaptic dysfunction and memory loss in AD (Wang X. et al., 2018).

Based on these studies, miR-124 is involved in the pathogenesis of AD by promoting Aβ production, enhancing tau phosphorylation, impairing synaptic function, and influencing the MG activation state. Upregulated miR-124 protects patients with AD from pathological damage by inhibiting Aβ production and reducing tau phosphorylation and synaptic loss, providing a potential strategy for treating AD.

### Parkinson’s Disease

Parkinson’s disease is a common NDD with a prevalence rate of approximately 1.7% among people over 65 years of age in China. Most PD cases are sporadic and less than 10% have a family history. The leading pathological change in PD is the degeneration of DA neurons in the midbrain, which leads to a significant reduction in the striatum DA. The exact etiology of this pathological change remains unclear. The miR-124 level was found to be decreased in the plasma of patients with PD, with an area under the receiver operating characteristic curve of miR-124 of 0.709 (95% CI 0.618–0.633, *p* < 0.05), indicating that miR-124 is associated with PD and a potential biomarker for PD diagnosis (Li et al., 2017). In addition, plasma miR-124-3p levels were decreased in patients with PD compared to those in healthy controls (Ravanidis et al., 2020).

miR-124 is involved in the pathogenesis of PD mainly through neuroinflammation and the regulation of autophagy (Angelopoulou et al., 2019). In a previous study, miR-124 was significantly downregulated in a 1-methyl-4-phenyl-1,2,3,6-tetrahydropyridine (MPTP)-induced mouse model of PD and suggested to inhibit neuroinflammation during PD development (Slota and Booth, 2019). The expression of sequestosome-1 (p62) and phospho-p38 (p-p38) mitogen-activated protein kinase was significantly increased in lipopolysaccharide-treated immortalized murine BV2 cells in an MPTP-induced mouse model of PD. Furthermore, exogenous delivery of miR-124 suppressed p62 and p-p38 expression and attenuated the activation of MG in MPTP-treated mice, indicating that miR-124 inhibits neuroinflammation during PD development by targeting p62, p38, and autophagy (Yao et al., 2019). miR-124-3p expression was decreased in MPP^+^-induced SH-SY5Y cells. miR-124-3p overexpression exerted protective effects by increasing cell viability and reducing cell apoptosis, caspase-3 activity, inflammatory factors TNF-α, and IL-1β levels as well as by attenuating MPP^+^-induced neuronal injury. In addition, miR-124-3p targets STAT3, mediating the neuroprotective effect (Geng et al., 2017). Mitogen-activated protein kinase 3 (MEKK3) expression was increased in an MPTP-induced mouse model of PD, thus promoting the activation of MG by regulating nuclear factor (NF)-κB expression. Exogenous delivery of miR-124 inhibited MEKK3 and p-p65 expression and attenuated the activation of MG in the substantia nigra pars compacta of MPTP-treated mice, indicating miR-124 can inhibit neuroinflammation during the development of PD by regulating the MEKK3/NF-κB signaling pathways (Yao et al., 2018).

miR-124 may be involved in the pathogenesis of PD through other mechanisms. miR-124-3p expression was downregulated in 6-hydroxydopamine (6-OHDA)-treated PC12 and SH-SY5Y cells. Direct targeting of annexinA5 by miR-124-3p enhanced the viability of 6-OHDA-treated PC12 or SH-SY5Y cells, which was associated with stimulation of the extracellular signal-regulated kinase pathway (Dong et al., 2018). A calpain-p25-mediated increase in cdk5 expression leads to dopaminergic neuronal death in human PD and MPTP-PD models. Additionally, an interaction between miR-124 with calpain 1 was identified in experiments using miR-124 target protector sequences; miR-124 overexpression attenuated the expression of calpain 1/p25/cdk5 proteins and improved cell survival. Furthermore, miR-124 was suggested to regulate the expression of calpain 1/p25/cdk5/pathway proteins in dopaminergic neurons (Kanagaraj et al., 2014). miR-124 also affected dopamine receptor expression, neuronal proliferation, and apoptosis in MPTP-induced mouse models of PD. Endothelin 2 has been identified as a target of miR-124; miR-124 overexpression promoted dopamine receptor expression and neuronal proliferation, and suppressed neuronal apoptosis by downregulating endothelin 2 *via* activation of the Hedgehog signaling pathway (Wang et al., 2019). Bim is a BH3-only protein shown to be involved in apoptosis of DA neurons in an MPTP model of PD, in addition to being a direct target of miR-124. Upregulation of miR-124 significantly reduced the loss of DA neurons in MPTP-treated mice by regulating Bim expression (Wang H. et al., 2016).

Furthermore, regulation of miR-124 expression is a promising strategy for PD treatment. In a previous study, miR-124 loaded nanoparticles (NPs) enhanced brain repair in PD. Intracerebral administration of miR-124 NPs increased the number of migrating neuroblasts and induced the migration of neurons into the lesioned striatum of 6-OHDA-treated mice. Thus, miR-124 NPs were considered as a potential new therapeutic approach for promoting endogenous brain repair mechanisms in NDDs (Saraiva et al., 2016b). In addition, miR-124 NPs were suggested to target both the MEKK3 and NF-κB pathways, and reduce inflammatory cytokine levels (Gan et al., 2019).

In general, the expression of miR-124 is down-regulated in various PD models, miR-124 serves as a protective factor in DA neurons in PD, and up-regulation of miR-124 can protect DA neurons in PD from injury via multiple mechanisms, including by inhibiting MG activation. Therefore, miR-125 may have a therapeutic role in PD.

### Huntington’s Disease

Huntington’s disease is an autosomal dominant genetic disease characterized by degenerative changes to the nervous system. Clinically, HD mainly manifests as dance-like movement, progressive cognitive decline, and mental symptoms. Abnormal amplification of the CAG repeat sequence at codon 17 downstream of the IT15 gene initiation codon is the main cause of the disease. In this NDD, altered miR-124 expression can lead to abnormal gene regulation. In HD striatal mutant STHdh (Q111)/Hdh (Q111) cells, miR-124 expression was shown to be downregulated. Cyclin A2 (CCNA2) was identified a target gene of miR-124, and increased miR-124 expression in R6/2 mice altered CCNA2 expression and the proportion of cells in S phase in the HD cell model, indicating that downregulation of miR-124 expression increased CCNA2 expression in HD and was involved in deregulation of the cell cycle in STHdh (Q111)/Hdh (Q111) cells (Das et al., 2013). In contrast, miR-124 overexpression exhibited therapeutic effects in an HD model. Exosome-based delivery of miR-124 to the striatum of R6/2 transgenic HD mice reduced REST expression. Although the treatment did not produce significant behavioral improvement, it offered possible therapeutic strategies from a pathophysiological perspective (Lee et al., 2017). In summary, based on current limited evidence, miR-124 appears to play a protective role in the progression of HD.

### Amyotrophic Lateral Sclerosis

Amyotrophic lateral sclerosis (ALS), the most common type of motor neuron disease, is a lethal NDD, the pathogenesis of which is not completely understood. The lesion involves both upper and lower motor neurons and is characterized by progressive limb weakness and muscle atrophy with pyramidal tract signs. Between 5% and 10% of patients with ALS have a family history; however, the number of discovered genes clearly associated with ALS remains very limited (Petrov et al., 2017). Protein misfolding and Cu/Zn superoxide dismutase 1 mutations are associated with inflammatory and neurotoxic pathways in ALS (Fan et al., 2020).

miR-124 expression was downregulated in the spinal cord and brainstem and upregulated in differentiated neural stem cells in G93A-superoxide dismutase 1 mice. Sox2 and Sox9 were identified as target genes of miR-124 and their protein levels showed opposite changes with miR-124 expression *in vivo* and *in vitro*. Thus, miR-124 was suggested to play an important role in astrocytic differentiation by targeting Sox2 and Sox9 in ALS transgenic mice (Zhou et al., 2018). Dysregulation of certain miRs can contribute to MG hyperactivation, persistent neuroinflammation, and abnormal macrophage polarization in the brain (Guo et al., 2019). Increased miR-124 expression caused persistent activation of NF-κB and matrix metalloproteinases 2 and 9, as well as upregulation of major histocompatibility complex-II, TNF-α, IL-1β, and inducible nitric oxide synthase gene expression, indicating induced M1 polarization. The data indicated that modulation of the inflammatory-associated miR-124 could determine early and late phenotypic alterations in the recipient N9-MG, providing a promising therapeutic approach for inhibiting MG activation and its associated effects in motor neuron degeneration (Pinto et al., 2017). In addition, MG-associated inflammatory biomarkers, such as NF-κB/Nlrp3-inflammasome and pro-inflammatory cytokines, were increased during the symptomatic stage of ALS, accompanied by upregulated miR-124 expression. These results highlight the role of miR-124 in activating MG and provide candidate miRs that may exert potential neuroprotective strategies in ALS therapy (Cunha et al., 2018).

## MicroRNA-124 and Multiple Sclerosis

Multiple sclerosis (MS) is an immune-mediated chronic inflammatory demyelinating disease of the CNS. Abnormal activation of cellular and humoral immunity eventually leads to loss of the myelin sheath in the CNS and damage to oligodendrocytes, some of which may involve axons and neurons (Musella et al., 2018). miR-124 was significantly increased in demyelinated hippocampi and cortices from postmortem MS brains and inversely correlated with memory performance (Dutta et al., 2013). However, miR-124 was significantly downregulated in monocytes from patients with progressive MS, indicating the complete loss of homeostatic monocyte function during the progressive phase of the disease (Amoruso et al., 2020).

Emerging evidence has indicated that neuroinflammation is an important contributor to MS and that miR-124 is involved in the pathogenesis of MS as a neuroinflammation regulator. Resveratrol shows potential as an effective therapeutic agent in experimental autoimmune encephalomyelitis, a murine model of MS. Resveratrol effectively decreased MS severity, including inflammation and CNS immune cell infiltration. Further investigations of the therapeutic mechanism showed that resveratrol could upregulate miR-124 expression and then suppress sphingosine kinase 1 expression (associated target gene of miR-124), indicating that upregulation of miR-124 can suppress neuroinflammation and halt cell-cycle progression in activated encephalitogenic T cells (Gandy et al., 2019).

## MicroRNA-124 and Peripheral Neuropathy

Neuropathic pain is caused by somatosensory dysfunction of the peripheral system and CNS and is associated with spontaneous pain, such as sensory disorders, paresthesia, hyperalgesia, and hypersensitivity. Although significant progress has been made in the pathogenesis and treatment of neuropathic pain, therapeutic results remain unsatisfactory (Scholz and Woolf, 2007; Van Hecke et al., 2014). Dysregulated expression of miRs plays key roles in neuropathic pain development. In a chemotherapy-induced peripheral neuropathic animal model, circulating miR-124 levels were increased, which correlated with axonal degeneration in both the dorsal root ganglion and sciatic nerve. Although these results cannot yet be applied in clinical practice, they provide positive evidence that circulating miR-124 can be used as a diagnostic biomarker for the early diagnosis of peripheral neuropathy (Peng et al., 2019). In the chronic constriction injury model of neuropathic pain in rats, miR-124 was upregulated; however, modulation of miRs did not appear to significantly contribute to changes in gene expression in the spinal cord in this chronic neuropathic pain model (Brandenburger et al., 2012).

miR-124-3p was dramatically downregulated in rats after chronic sciatic nerve injury; in contrast, miR-124-3p overexpression reduced the levels of inflammatory cytokines, such as interleukin (IL)-1β, IL-6, and TNF-α, and inhibited mechanical allodynia and heat hyperalgesia. In addition, EZH2 was identified as a target of miR-124-3p. Therefore, miR-124-3p may promote neuroinflammation and neuropathic pain by targeting EZH2 as a promising therapeutic strategy for neuropathic pain (Zhang et al., 2019). Furthermore, miR-124 effectively reversed established hyperalgesia in morphine-induced persistent sensitization models. The mechanism may be associated with targeting of Toll-like receptor signals, highlighting the therapeutic potential of miR-124 in treating peripheral neuropathy (Grace et al., 2018).

## MicroRNA-124 and Epilepsy

Epilepsy is a chronic neurological disorder characterized by recurrent seizures and caused by abnormal and synchronized firing of neurons in the brain (Alves et al., 2019). miR-124 expression was downregulated in epileptic patients and drug-induced epileptic rats. In two drug-induced rat epilepsy models, intra-hippocampal administration of miR-124 reduced the severity of epilepsy and prolonged the incubation period. cAMP-response element-binding protein1 (CREB1) was identified as a target gene of miR-124, and miR-124 overexpression repressed CREB1 expression, which is a key regulator in epileptogenesis, indicating miR-124 is involved in the pathogenesis of epilepsy by regulating CREB1 expression (Wang W. et al., 2016). miR-124 expression is dynamic during the three stages of mesial temporal lobe epilepsy (MTLE) development. miR-124 was significantly upregulated in hippocampal tissues in the acute and chronic stages of MTLE but nearly returned to normal in the latent stage, indicating that this miRNA can be used as a biomarker for MTLE diagnosis and therapeutic target for anticonvulsant drugs (Peng et al., 2013).

miR-124 expression was found to be low in refractory epilepsy rats, and upregulation of the miR-124 level increased the seizure interval, improved the cognitive function of rats, and promoted PI3K and AKT expression. Thus, miR-124 may play a protective role in temporal lobe epilepsy by promoting PI3K/Akt signaling pathway (Wang R. et al., 2020). miR-124 expression was also downregulated in status epilepticus rats. miR-124 upregulation reduced neuron-restrictive silencer factor expression and inhibited MG activation and inflammatory cytokine expression (Brennan et al., 2016).

Most studies support that miR-124 show low expression in epilepsy models, particularly in the acute phase of epilepsy or status epilepticus models. Upregulating the expression of miR-124 can protect against neuronal impairment in epilepsy, and studies have suggested that the protective effect of miR-124 can be achieved by inhibiting the inflammatory response in the CNS.

## MicroRNA-124 and Central Nervous System Trauma

### Traumatic Brain Injury

Traumatic brain injury (TBI) is defined as traumatic structural injury and/or brain dysfunction caused by external forces and is clinically characterized by a loss of consciousness, memory loss, altered mental state, neurological dysfunction, and intracranial damage (Ruff et al., 2009). miR-124-3p levels were elevated at 2 days post-TBI in both the blood and plasma of patients; an elevated plasma miR-124-3p level 2 days post-TBI was positively correlated with larger lesion area at the chronic time point in TBI models (Vuokila et al., 2020). Furthermore, miR-124-3p was detected only in patients with severe TBI (Schindler et al., 2020). The miR-124-3p level was elevated in the serum of patients with TBI (O’connell et al., 2020). However, miR-124-3p was downregulated in post-TBI hippocampal pathologies in experimental models and in humans (Vuokila et al., 2018).

Neuroinflammation is the characteristic pathological change occurring during acute nerve injury after TBI. Inhibiting the excessive inflammatory response is crucial for improving the prognosis of the nervous system. miR-124-3p was shown to promote anti-inflamed M2 polarization in MG, and an increase in the level of miR-124-3p in MG exosomes after TBI inhibited neuroinflammation and contributed to neurite outgrowth by facilitating their transfer into neurons. miR-124-3p exerted these effects by targeting PDE4B, thus inhibiting mTOR signaling activity (Huang et al., 2018). In addition, exo-miR-124 treatment was suggested to promote M2 polarization of MG and improve hippocampal neurogenesis and functional recovery after TBI (Yang et al., 2019). MG exo-miR-124-3p may also inhibit neuronal autophagy and protect against nerve injury by facilitating their transfer into neurons for the treatment of nerve injury after TBI (Li et al., 2019).

### Spinal Cord Injury

Spinal cord injury (SCI) is a highly disabling injury to the CNS caused by trauma. Destruction of the spinal cord (SC) structure can lead to an inflammatory response, immune injury, and other mechanisms in the damaged SC tissue, resulting in spinal cord dysfunction. miR-124 expression in neurons was shown to be significantly decreased within 7 days after SCI and may reflect the severity of SCI (Zhao et al., 2015).

miR-124 was decreased in a rat model of SCI. GTP-cyclohydrolase 1, a target gene of miR-124, plays an important role in SCI-induced neuronal apoptosis. miR-124 overexpression inhibited neuronal apoptosis in SCI by modulating GTP-cyclohydrolase 1 expression (Yuan et al., 2019). Tal1 is also a potential target gene of miR-124, and its downregulation promoted the proliferation of neuronal precursor cells and inhibited their differentiation, indicating miR-124 can mediate SCI repair by altering the expression of various mRNAs in rats (Wang J. et al., 2020). miR-124 was notably downregulated in SCI rats; miR-124 overexpression improved functional recovery and decreased the lesion size in SCI rats. BAX, an apoptosis regulator, is a target of miR-124; thus, miR-124 suppressed neuronal cell apoptosis in an SCI rat model by inhibiting BAX expression (Xu et al., 2019). miR-124 was shown to target pyridoxal kinase to accelerate the differentiation of bone marrow mesenchymal stem cells into neurocytes and promote SCI repair (Song et al., 2017). Exosomal miR-124-3p (exo-miR-124-3p) derived from bone marrow mesenchymal stem cells attenuated nerve injury induced by SC ischemia-reperfusion injury by regulating Ern1 and M2 macrophage polarization (Li et al., 2020). In addition, PI3K/AKT/NF-κB signaling cascades were involved in modulating MG via exo-miR-124-3p (Jiang et al., 2020).

Despite these different conclusions, the miR-124 expression level in the peripheral blood of patients with SCI showed an increasing trend and was positively correlated with the severity of SCI. In animal models, miR-124 expression was mainly downregulated, and miR-124 overexpression may play a protective role by inhibiting inflammation and neuronal apoptosis and by promoting nerve regeneration.

## Discussion and Perspectives

Herein, we reviewed the role of miR-124 in neurological disorders and its mechanism in regulating pathophysiological processes. miR-124 is involved in the pathogenesis of neurological disorders through various mechanisms, mainly post-transcriptional regulation of gene expression, glial cell activation, and neuroinflammatory response. The feasibility of using miR-124 as a circulating biomarker and therapeutic target in the diagnosis of neurological disorders was also reviewed.

miR-124 is highly conserved and is among the most abundant miRs specifically expressed in the CNS. In addition, miR-124 is closely associated with nervous system development, injury, and repair (Han et al., 2019). Changes in miR-124 expression levels may play a key role in the occurrence of various neurological disorders. A review of previous studies revealed the extensive roles of miR-124 in the pathology of these disorders. Notably, miR-124 expression is downregulated during the acute phase of most neurological disorders, such as CVD, NDDs, neurological tumors, and TBI. However, this expression level changes dynamically. For example, the level is significantly downregulated in the early onset of AIS and, as the disease develops, it gradually returns to normal. Although the mechanism of changes in the miR-124 expression level is unclear, most studies revealed that a normal miR-124 level has a protective effect on the nervous system. Low miR-124 expression is correlated with the occurrence of neurological disorders, and miR-124 overexpression can protect neurons or have a therapeutic effect on diseases. miR-124 is abundantly expressed in the CNS and involved in the pathogenesis of various neurological disorders. The main function of miR-124 is regulating gene expression at the post-transcriptional level. miR-124 directly regulates many key pathogenic genes of the nervous system, such as the target gene BACE1, a critical enzyme that controls the production of Aβ. In addition, miR-124 is involved in activating MG in many neurological disorders. For example, miR-124 is significantly increased in M2-polarized MG in CVD and TBI and promotes the activation of MG by regulating NF-κB expression in PD. Furthermore, miR-124 participates in various neuroinflammation reactions; miR-124 inhibits neuroinflammation during the development of PD by regulating the MEKK3/NF-κB signaling pathways, suppressing neuroinflammation, and halting cell-cycle progression in activated encephalitogenic T cells in MS. Understanding the involvement of miR-124 in neurological disorders may facilitate the clinical application of miR-124 as a diagnostic biomarker and therapeutic target.

Changes in peripheral blood miR-124 levels may become a biomarker of neurological disorders. The miR-124-3p level was significantly increased in AIS patients and associated with poor prognosis. In addition, the miR-124-3p level in patients with ICH was higher than in patients with AIS, indicating that the level of this miRNA has diagnostic value for CVDs and can be used to distinguish between ischemic and hemorrhagic CVDs. Among NDDs, the miR-124 levels in the plasma of patients with PD were significantly lower than those in healthy controls. Furthermore, the miR-124 expression level in the monocytes of patients with MS was significantly decreased; this decline was also observed in patients with epilepsy. Although the expression level differs from that in some animal models, miR-124 expression in human patients may better reflect the actual clinical situation. Considering the wide and dynamic changes in miR-124 in neurological disorders, further clinical data are needed to determine its usefulness as a biomarker, and the specificity of miR-124 should be quantified in different neurological disorders. Furthermore, changes in the miR-124 expression level in the peripheral circulation differ from those in the brain tissue even in the same neurological disorders, as expected. The blood-brain barrier creates very different biochemical conditions between the brain and blood. Different tissue-specific miR-124 levels may be caused by damage to the blood-brain barrier. Changes in miR-124 levels in the brain may be useful for targeted treatment.

The results of mechanistic studies showed that miR-124 exerted a protective effect on the nervous system *via* various mechanisms such as post-transcriptional regulation of gene expression, regulation of glial cell transformation to an anti-inflammatory phenotype, and inhibition of the neuroinflammatory response. However, miR-124 has several potential side effects. Since miR-124 has multiple target genes, further research is needed to confirm whether miR-124 interacts with other genes to produce unpredictable effects while exerting its therapeutic activities.

Virus-based delivery systems are highly efficient and can effectively deliver miRNAs to target cells. Intracranial injection of adeno-associated virus expressing miR-124-3p into AD mice significantly reduced Aβ deposition and improved the cognitive function of AD mice (Yao et al., 2019). Non-viral delivery systems are widely used in clinical studies because of their variety and relative safety (Yin et al., 2014). miR-124-loaded polymeric NPs were constructed to treat a 6-OHDA-challenged mouse model of PD, resulting in improved motor function (Saraiva et al., 2016a). Exosomes exhibit a high level of miR-124 expression (exosome-miR-124) which decreased REST target gene expression in R6/2 transgenic HD mice; this gene is involved in multiple links in the pathogenesis of HD (Lee et al., 2017). Although the *in vivo* delivery of miRs by adeno-associated virus, NPs, and exosomes has been successfully tested in various animal models, further *in vivo* experiments are needed before clinical application.

## Conclusion

miR-124 plays an important role in the occurrence and development of various neurological disorders and may be useful as a biomarker for the diagnosis and prognosis of these disorders. However, additional human studies are needed to verify its clinical application prospects. Understanding the functional role of miR-124 in regulating pathological mechanisms and other regulatory pathways in different nerve injuries, cell types, and disease stages will facilitate the use of miR-124 as a therapeutic target for neurological disorders.

## Author Contributions

JZ and JW performed the literature search. JZ drafted the manuscript. JW critically revised the manuscript. All authors contributed to the article and approved the submitted version.

## Conflict of Interest

The authors declare that the research was conducted in the absence of any commercial or financial relationships that could be construed as a potential conflict of interest.

## Publisher’s Note

All claims expressed in this article are solely those of the authors and do not necessarily represent those of their affiliated organizations, or those of the publisher, the editors and the reviewers. Any product that may be evaluated in this article, or claim that may be made by its manufacturer, is not guaranteed or endorsed by the publisher.

## References

[B1] Aguzzi A. Barres B. A. Bennett M. L. (2013). Microglia: scapegoat, saboteur, or something else? *Science* 339 156–161. 10.1126/science.1227901 23307732 PMC4431634

[B2] Alves M. Kenny A. De Leo G. Beamer E. H. Engel T. (2019). Tau Phosphorylation in a Mouse Model of Temporal Lobe Epilepsy. *Front Aging Neurosci* 11:308. 10.3389/fnagi.2019.00308 31780921 PMC6861366

[B3] Amoruso A. Blonda M. Gironi M. Grasso R. Di Francescantonio V. Scaroni F. (2020). Immune and central nervous system-related miRNAs expression profiling in monocytes of multiple sclerosis patients. *Sci Rep* 10 6125. 10.1038/s41598-020-63282-3 32273558 PMC7145856

[B4] Angelopoulou E. Paudel Y. N. Piperi C. (2019). miR-124 and Parkinson’s disease: A biomarker with therapeutic potential. *Pharmacol Res* 150 104515. 10.1016/j.phrs.2019.104515 31707035

[B5] Ayaz M. Sadiq A. Junaid M. Ullah F. Ovais M. Ullah I. (2019). Flavonoids as Prospective Neuroprotectants and Their Therapeutic Propensity in Aging Associated Neurological Disorders. *Front Aging Neurosci* 11:155. 10.3389/fnagi.2019.00155 31293414 PMC6606780

[B6] Badacz R. Kleczyński P. Legutko J. Żmudka K. Gacoń J. Przewłocki T. (2021). Expression of miR-1-3p, miR-16-5p and miR-122-5p as Possible Risk Factors of Secondary Cardiovascular Events. *Biomedicines* 9 1055. 10.3390/biomedicines9081055 34440258 PMC8391895

[B7] Bahlakeh G. Gorji A. Soltani H. Ghadiri T. (2021). MicroRNA alterations in neuropathologic cognitive disorders with an emphasis on dementia: Lessons from animal models. *J Cell Physiol* 236 806–823. 10.1002/jcp.29908 32602584

[B8] Brandenburger T. Castoldi M. Brendel M. Grievink H. Schlösser L. Werdehausen R. (2012). Expression of spinal cord microRNAs in a rat model of chronic neuropathic pain. *Neurosci Lett* 506 281–286. 10.1016/j.neulet.2011.11.023 22138088

[B9] Brennan G. P. Dey D. Chen Y. Patterson K. P. Magnetta E. J. Hall A. M. (2016). Dual and Opposing Roles of MicroRNA-124 in Epilepsy Are Mediated through Inflammatory and NRSF-Dependent Gene Networks. *Cell Rep* 14 2402–2412. 10.1016/j.celrep.2016.02.042 26947066 PMC4794429

[B10] Broderick J. P. (1993). Stroke trends in Rochester, Minnesota, during 1945 to 1984. *Ann Epidemiol* 3 476–479. 10.1016/1047-2797(93)90099-P 8167821

[B11] Burke T. A. Venketasubramanian R. N. (2006). The epidemiology of stroke in the East Asian region: a literature-based review. *Int J Stroke* 1 208–215. 18706018 10.1111/j.1747-4949.2006.00060.x

[B12] Chen X. Jiang M. Li H. Wang Y. Shen H. Li X. (2020). CX3CL1/CX3CR1 axis attenuates early brain injury via promoting the delivery of exosomal microRNA-124 from neuron to microglia after subarachnoid hemorrhage. *J Neuroinflammation* 17 209. 10.1186/s12974-020-01882-6 32664984 PMC7362528

[B13] Choe N. Kwon D. H. Shin S. Kim Y. S. Kim Y. K. Kim J. (2017). The microRNA miR-124 inhibits vascular smooth muscle cell proliferation by targeting S100 calcium-binding protein A4 (S100A4). *FEBS Lett* 591 1041–1052. 10.1007/s12035-017-0631-2 28235243

[B14] Cunha C. Santos C. Gomes C. Fernandes A. Correia A. M. Sebastião A. M. (2018). Downregulated Glia Interplay and Increased miRNA-155 as Promising Markers to Track ALS at an Early Stage. *Mol Neurobiol* 55 4207–4224. 28612258 10.1007/s12035-017-0631-2

[B15] Das E. Jana N. R. Bhattacharyya N. P. (2013). MicroRNA-124 targets CCNA2 and regulates cell cycle in STHdh(Q111)/Hdh(Q111) cells. *Biochem Biophys Res Commun* 437 217–224. 10.1016/j.bbrc.2013.06.041 23796713

[B16] Devaux Y. Dankiewicz J. Salgado-Somoza A. Stammet P. Collignon O. Gilje P. (2016). Association of Circulating MicroRNA-124-3p Levels With Outcomes After Out-of-Hospital Cardiac Arrest: A Substudy of a Randomized Clinical Trial. *JAMA Cardiol* 1 305–313. 10.1001/jamacardio.2016.0480 27438111

[B17] Dong R. F. Zhang B. Tai L. W. Liu H. M. Shi F. K. Liu N. N. (2018). The Neuroprotective Role of MiR-124-3p in a 6-Hydroxydopamine-Induced Cell Model of Parkinson’s Disease via the Regulation of ANAX5. *J Cell Biochem* 119 269–277. 28543594 10.1002/jcb.26170

[B18] Dutta R. Chomyk A. M. Chang A. Ribaudo M. V. Deckard S. A. Doud M. K. (2013). Hippocampal demyelination and memory dysfunction are associated with increased levels of the neuronal microRNA miR-124 and reduced AMPA receptors. *Ann Neurol* 73 637–645. 10.1002/ana.23860 23595422 PMC3679350

[B19] Fan W. Liang C. Ou M. Zou T. Sun F. Zhou H. (2020). MicroRNA-146a Is a Wide-Reaching Neuroinflammatory Regulator and Potential Treatment Target in Neurological Diseases. *Front Mol Neurosci* 13:90. 10.3389/fnmol.2020.00090 32581706 PMC7291868

[B20] Gan L. Li Z. Lv Q. Huang W. (2019). Rabies virus glycoprotein (RVG29)-linked microRNA-124-loaded polymeric nanoparticles inhibit neuroinflammation in a Parkinson’s disease model. *Int J Pharm* 567 118449. 10.1016/j.ijpharm.2019.118449 31226473

[B21] Gandy K. A. O. Zhang J. Nagarkatti P. Nagarkatti M. (2019). Resveratrol (3, 5, 4′-Trihydroxy-trans-Stilbene) Attenuates a Mouse Model of Multiple Sclerosis by Altering the miR-124/Sphingosine Kinase 1 Axis in Encephalitogenic T Cells in the Brain. *J Neuroimmune Pharmacol* 14 462–477. 30941623 10.1007/s11481-019-09842-5 PMC6900929

[B22] Gascon E. Lynch K. Ruan H. Almeida S. Verheyden J. M. Seeley W. W. (2014). Alterations in microRNA-124 and AMPA receptors contribute to social behavioral deficits in frontotemporal dementia. *Nat Med* 20 1444–1451. 10.1038/nm.3717 25401692 PMC4257887

[B23] Ge X. Guo M. Hu T. Li W. Huang S. Yin Z. (2020). Increased Microglial Exosomal miR-124-3p Alleviates Neurodegeneration and Improves Cognitive Outcome after rmTBI. *Mol Ther* 28 503–522. 31843449 10.1016/j.ymthe.2019.11.017 PMC7001001

[B24] Geng L. Liu W. Chen Y. (2017). miR-124-3p attenuates MPP(+)-induced neuronal injury by targeting STAT3 in SH-SY5Y cells. *Exp Biol Med (Maywood)* 242 1757–1764. 10.1177/1535370217734492 28958159 PMC5714150

[B25] Grace P. M. Strand K. A. Galer E. L. Maier S. F. Watkins L. R. (2018). MicroRNA-124 and microRNA-146a both attenuate persistent neuropathic pain induced by morphine in male rats. *Brain Res* 1692 9–11. 10.1016/j.brainres.2018.04.038 29723521 PMC5976546

[B26] Guo Y. Hong W. Wang X. Zhang P. Körner H. Tu J. (2019). MicroRNAs in Microglia: How do MicroRNAs Affect Activation, Inflammation, Polarization of Microglia and Mediate the Interaction Between Microglia and Glioma? *Front Mol Neurosci* 12:125. 10.3389/fnmol.2019.00125 31133802 PMC6522842

[B27] Hamzei Taj S. Kho W. Riou A. Wiedermann D. Hoehn M. (2016). MiRNA-124 induces neuroprotection and functional improvement after focal cerebral ischemia. *Biomaterials* 91 151–165. 10.1016/j.biomaterials.2016.03.025 27031810

[B28] Han D. Dong X. Zheng D. Nao J. (2019). MiR-124 and the Underlying Therapeutic Promise of Neurodegenerative Disorders. *Front Pharmacol* 10:1555. 10.3389/fphar.2019.01555 32009959 PMC6978711

[B29] He X. W. Shi Y. H. Liu Y. S. Li G. F. Zhao R. Hu Y. (2019). Increased plasma levels of miR-124-3p, miR-125b-5p and miR-192-5p are associated with outcomes in acute ischaemic stroke patients receiving thrombolysis. *Atherosclerosis* 289 36–43. 10.1016/j.atherosclerosis.2019.08.002 31450012

[B30] Huang S. Ge X. Yu J. Han Z. Yin Z. Li Y. (2018). Increased miR-124-3p in microglial exosomes following traumatic brain injury inhibits neuronal inflammation and contributes to neurite outgrowth via their transfer into neurons. *Faseb j* 32 512–528. 10.1096/fj.201700673r 28935818

[B31] Ji Q. Ji Y. Peng J. Zhou X. Chen X. Zhao H. (2016). Increased Brain-Specific MiR-9 and MiR-124 in the Serum Exosomes of Acute Ischemic Stroke Patients. *PLoS One* 11:e0163645. 10.1371/journal.pone.0163645 27661079 PMC5035015

[B32] Jiang D. Gong F. Ge X. Lv C. Huang C. Feng S. (2020). Neuron-derived exosomes-transmitted miR-124-3p protect traumatically injured spinal cord by suppressing the activation of neurotoxic microglia and astrocytes. *J Nanobiotechnology* 18 105. 10.1186/s12951-020-00665-8 32711535 PMC7382861

[B33] Kanagaraj N. Beiping H. Dheen S. T. Tay S. S. (2014). Downregulation of miR-124 in MPTP-treated mouse model of Parkinson’s disease and MPP iodide-treated MN9D cells modulates the expression of the calpain/cdk5 pathway proteins. *Neuroscience* 272 167–179. 10.1016/j.neuroscience.2014.04.039 24792712

[B34] Kang Q. Xiang Y. Li D. Liang J. Zhang X. Zhou F. (2017). MiR-124-3p attenuates hyperphosphorylation of Tau protein-induced apoptosis via caveolin-1-PI3K/Akt/GSK3β pathway in N2a/APP695swe cells. *Oncotarget* 8 24314–24326. 28186985 10.18632/oncotarget.15149 PMC5421849

[B35] Lagos-Quintana M. Rauhut R. Yalcin A. Meyer J. Lendeckel W. Tuschl T. (2002). Identification of tissue-specific microRNAs from mouse. *Curr Biol* 12 735–739. 10.1016/S0960-9822(02)00809-6 12007417

[B36] Landgraf P. Rusu M. Sheridan R. Sewer A. Iovino N. Aravin A. (2007). A mammalian microRNA expression atlas based on small RNA library sequencing. *Cell* 129 1401–1414. 17604727 10.1016/j.cell.2007.04.040 PMC2681231

[B37] Lee S. T. Im W. Ban J. J. Lee M. Jung K. H. Lee S. K. (2017). Exosome-Based Delivery of miR-124 in a Huntington’s Disease Model. *J Mov Disord* 10 45–52. 10.14802/jmd.16054 28122430 PMC5288667

[B38] Leung L. Y. Chan C. P. Leung Y. K. Jiang H. L. Abrigo J. M. Wang De F. (2014). Comparison of miR-124-3p and miR-16 for early diagnosis of hemorrhagic and ischemic stroke. *Clin Chim Acta* 433 139–144. 24650689 10.1016/j.cca.2014.03.007

[B39] Li D. Huang S. Yin Z. Zhu J. Ge X. Han Z. (2019). Increases in miR-124-3p in Microglial Exosomes Confer Neuroprotective Effects by Targeting FIP200-Mediated Neuronal Autophagy Following Traumatic Brain Injury. *Neurochem Res* 44 1903–1923. 10.1007/s11064-019-02825-1 31190315

[B40] Li N. Pan X. Zhang J. Ma A. Yang S. Ma J. (2017). Plasma levels of miR-137 and miR-124 are associated with Parkinson’s disease but not with Parkinson’s disease with depression. *Neurol Sci* 38 761–767. 10.1007/s10072-017-2841-9 28181066

[B41] Li R. Zhao K. Ruan Q. Meng C. Yin F. (2020). Bone marrow mesenchymal stem cell-derived exosomal microRNA-124-3p attenuates neurological damage in spinal cord ischemia-reperfusion injury by downregulating Ern1 and promoting M2 macrophage polarization. *Arthritis Res Ther* 22 75. 10.1186/s13075-020-2146-x 32272965 PMC7146970

[B42] Li Z. Song Y. He T. Wen R. Li Y. Chen T. (2021). M2 microglial small extracellular vesicles reduce glial scar formation via the miR-124/STAT3 pathway after ischemic stroke in mice. *Theranostics* 11 1232–1248. 10.7150/thno.48761 33391532 PMC7738903

[B43] Liu Y. Zhang J. Han R. Liu H. Sun D. Liu X. (2015). Downregulation of serum brain specific microRNA is associated with inflammation and infarct volume in acute ischemic stroke. *J Clin Neurosci* 22 291–295. 10.1016/j.jocn.2014.05.042 25257664

[B44] Louw A. M. Kolar M. K. Novikova L. N. Kingham P. J. Wiberg M. Kjems J. (2016). Chitosan polyplex mediated delivery of miRNA-124 reduces activation of microglial cells in vitro and in rat models of spinal cord injury. *Nanomedicine* 12 643–653. 26582736 10.1016/j.nano.2015.10.011

[B45] Makeyev E. V. Zhang J. Carrasco M. A. Maniatis T. (2007). The MicroRNA miR-124 promotes neuronal differentiation by triggering brain-specific alternative pre-mRNA splicing. *Mol Cell* 27 435–448. 10.1016/j.molcel.2007.07.015 17679093 PMC3139456

[B46] Musella A. Gentile A. Rizzo F. R. De Vito F. Fresegna D. Bullitta S. (2018). Interplay Between Age and Neuroinflammation in Multiple Sclerosis: Effects on Motor and Cognitive Functions. *Front Aging Neurosci* 10:238. 10.3389/fnagi.2018.00238 30135651 PMC6092506

[B47] O’connell G. C. Smothers C. G. Winkelman C. (2020). Bioinformatic analysis of brain-specific miRNAs for identification of candidate traumatic brain injury blood biomarkers. *Brain Inj* 34 965–974. 10.1080/02699052.2020.1764102 32497449

[B48] Peng J. Omran A. Ashhab M. U. Kong H. Gan N. He F. (2013). Expression patterns of miR-124, miR-134, miR-132, and miR-21 in an immature rat model and children with mesial temporal lobe epilepsy. *J Mol Neurosci* 50 291–297. 10.1007/s12031-013-9953-3 23315173

[B49] Peng Q. Bakulski K. M. Nan B. Park S. K. (2017). Cadmium and Alzheimer’s disease mortality in U.S. adults: Updated evidence with a urinary biomarker and extended follow-up time. *Environ Res* 157 44–51. 10.1016/j.envres.2017.05.011 28511080 PMC5513740

[B50] Peng Q. Mechanic J. Shoieb A. Pardo I. D. Schaevitz L. Fenyk-Melody J. (2019). Circulating microRNA and automated motion analysis as novel methods of assessing chemotherapy-induced peripheral neuropathy in mice. *PLoS One* 14:e0210995. 10.1371/journal.pone.0210995 30677061 PMC6345499

[B51] Periyasamy P. Liao K. Kook Y. H. Niu F. Callen S. E. Guo M. L. (2018). Cocaine-Mediated Downregulation of miR-124 Activates Microglia by Targeting KLF4 and TLR4 Signaling. *Mol Neurobiol* 55 3196–3210. 10.1007/s12035-017-0584-5 28478506 PMC5673594

[B52] Petrov D. Mansfield C. Moussy A. Hermine O. (2017). ALS Clinical Trials Review: 20 Years of Failure. Are We Any Closer to Registering a New Treatment? *Front Aging Neurosci* 9:68. 10.3389/fnagi.2017.00068 28382000 PMC5360725

[B53] Pinto S. Cunha C. Barbosa M. Vaz A. R. Brites D. (2017). Exosomes from NSC-34 Cells Transfected with hSOD1-G93A Are Enriched in miR-124 and Drive Alterations in Microglia Phenotype. *Front Neurosci* 11:273. 10.3389/fnins.2017.00273 28567000 PMC5434170

[B54] Ponomarev E. D. Veremeyko T. Barteneva N. Krichevsky A. M. Weiner H. L. (2011). MicroRNA-124 promotes microglia quiescence and suppresses EAE by deactivating macrophages via the C/EBP-α-PU.1 pathway. *Nat Med* 17 64–70. 10.1038/nm.2266 21131957 PMC3044940

[B55] Rainer T. H. Leung L. Y. Chan C. P. Y. Leung Y. K. Abrigo J. M. Wang D. (2016). Plasma miR-124-3p and miR-16 concentrations as prognostic markers in acute stroke. *Clin Biochem* 49 663–668. 26968104 10.1016/j.clinbiochem.2016.02.016

[B56] Ravanidis S. Bougea A. Papagiannakis N. Koros C. Simitsi A. M. Pachi I. (2020). Validation of differentially expressed brain-enriched microRNAs in the plasma of PD patients. *Ann Clin Transl Neurol* 7 1594–1607. 32860338 10.1002/acn3.51146 PMC7480914

[B57] Ruff R. M. Iverson G. L. Barth J. T. Bush S. S. Broshek D. K. (2009). Recommendations for diagnosing a mild traumatic brain injury: a National Academy of Neuropsychology education paper. *Arch Clin Neuropsychol* 24 3–10. 10.1093/arclin/acp006 19395352

[B58] Santalucia P. (2008). Intracerebral hemorrhage: medical treatment. *Neurol Sci* 29 S271–S273. 10.1007/s10072-008-0961-y 18690516

[B59] Saraiva C. Ferreira L. Bernardino L. (2016a). Traceable microRNA-124 loaded nanoparticles as a new promising therapeutic tool for Parkinson’s disease. *Neurogenesis (Austin)* 3 e1256855. 28405588 10.1080/23262133.2016.1256855 PMC5384609

[B60] Saraiva C. Paiva J. Santos T. Ferreira L. Bernardino L. (2016b). MicroRNA-124 loaded nanoparticles enhance brain repair in Parkinson’s disease. *J Control Release* 235 291–305. 10.1016/j.jconrel.2016.06.005 27269730

[B61] Schindler C. R. Woschek M. Vollrath J. T. Kontradowitz K. Lustenberger T. Störmann P. (2020). miR-142-3p Expression Is Predictive for Severe Traumatic Brain Injury (TBI) in Trauma Patients. *Int J Mol Sci* 21 5381. 10.3390/ijms21155381 PMC7432828 32751105

[B62] Scholz J. Woolf C. J. (2007). The neuropathic pain triad: neurons, immune cells and glia. *Nat Neurosci* 10 1361–1368. 10.1038/nn1992 17965656

[B63] Selkoe D. J. (2002). Alzheimer’s disease is a synaptic failure. *Science* 298 789–791. 10.1126/science.1074069 12399581

[B64] Serpe C. Monaco L. Relucenti M. Iovino L. Familiari P. Scavizzi F. (2021). ^∗^Microglia-Derived Small Extracellular Vesicles Reduce Glioma Growth by Modifying Tumor Cell Metabolism and Enhancing Glutamate Clearance through miR-124. *Cells* 10. 10.3390/cells10082066 PMC8393731 34440835

[B65] Slota J. A. Booth S. A. (2019). MicroRNAs in Neuroinflammation: Implications in Disease Pathogenesis, Biomarker Discovery and Therapeutic Applications. *Noncoding RNA* 5 35. 10.3390/ncrna5020035 31022830 PMC6632112

[B66] Song G. J. Suk K. (2017). Pharmacological Modulation of Functional Phenotypes of Microglia in Neurodegenerative Diseases. *Front Aging Neurosci* 9:139. 10.3389/fnagi.2017.00139 28555105 PMC5430023

[B67] Song J. L. Zheng W. Chen W. Qian Y. Ouyang Y. M. Fan C. Y. (2017). Lentivirus-mediated microRNA-124 gene-modified bone marrow mesenchymal stem cell transplantation promotes the repair of spinal cord injury in rats. *Exp Mol Med* 49 e332. 10.1038/emm.2017.48 28524176 PMC5454445

[B68] Sonntag K. C. Woo T. U. Krichevsky A. M. (2012). Converging miRNA functions in diverse brain disorders: a case for miR-124 and miR-126. *Exp Neurol* 235 427–435. 22178324 10.1016/j.expneurol.2011.11.035 PMC3335933

[B69] Sun M. Hou X. Ren G. Zhang Y. Cheng H. (2019). Dynamic changes in miR-124 levels in patients with acute cerebral infarction. *Int J Neurosci* 129 649–653. 10.1080/00207454.2018.1513931 30124350

[B70] Treiber T. Treiber N. Meister G. (2012). Regulation of microRNA biogenesis and function. *Thromb Haemost* 107 605–610. 22318703 10.1160/TH11-12-0836

[B71] Uddin M. S. Stachowiak A. Mamun A. A. Tzvetkov N. T. Takeda S. Atanasov A. G. (2018). Autophagy and Alzheimer’s Disease: From Molecular Mechanisms to Therapeutic Implications. *Front Aging Neurosci* 10:04. 10.3389/fnagi.2018.00004 29441009 PMC5797541

[B72] Van Hecke O. Austin S. K. Khan R. A. Smith B. H. Torrance N. (2014). Neuropathic pain in the general population: a systematic review of epidemiological studies. *Pain* 155 654–662. 10.1016/j.pain.2013.11.013 24291734

[B73] Volný O. Kašičková L. Coufalová D. Cimflová P. Novák J. (2015). microRNAs in Cerebrovascular Disease. *Adv Exp Med Biol* 888 155–195. 10.1007/978-3-319-22671-2_9 26663183

[B74] Vuokila N. Das Gupta S. Huusko R. Tohka J. Puhakka N. Pitkänen A. (2020). Elevated Acute Plasma miR-124-3p Level Relates to Evolution of Larger Cortical Lesion Area after Traumatic Brain Injury. *Neuroscience* 433 21–35. 32142864 10.1016/j.neuroscience.2020.02.045

[B75] Vuokila N. Lukasiuk K. Bot A. M. Van Vliet E. A. Aronica E. Pitkänen A. (2018). miR-124-3p is a chronic regulator of gene expression after brain injury. *Cell Mol Life Sci* 75 4557–4581. 30155647 10.1007/s00018-018-2911-z PMC11105702

[B76] Wang H. Ye Y. Zhu Z. Mo L. Lin C. Wang Q. (2016). MiR-124 Regulates Apoptosis and Autophagy Process in MPTP Model of Parkinson’s Disease by Targeting to Bim. *Brain Pathol* 26 167–176. 10.1111/bpa.12267 25976060 PMC8029438

[B77] Wang W. Wang X. Chen L. Zhang Y. Xu Z. Liu J. (2016). The microRNA miR-124 suppresses seizure activity and regulates CREB1 activity. *Expert Rev Mol Med* 18 e4. 10.1017/erm.2016.3 26996991 PMC4836211

[B78] Wang J. Li H. Chen L. Dong J. Yang J. Gong Z. (2020). mRNA Profiling for miR-124-mediated Repair in Spinal Cord Injury. *Neuroscience* 438 158–168. 10.1016/j.neuroscience.2020.05.013 32413396

[B79] Wang R. An X. Zhao S. (2020). Effect of miR-124 on PI3K/Akt signal pathway in refractory epilepsy rats. *Cell Mol Biol (Noisy-le-grand)* 66 146–152. 10.14715/cmb/2020.66.2.24 32415942

[B80] Wang J. Wang W. Zhai H. (2019). MicroRNA-124 Enhances Dopamine Receptor Expression and Neuronal Proliferation in Mouse Models of Parkinson’s Disease via the Hedgehog Signaling Pathway by Targeting EDN2. *Neuroimmunomodulation* 26 174–187. 10.1159/000501339 31454817

[B81] Wang X. Liu D. Huang H. Z. Wang Z. H. Hou T. Y. Yang X. (2018). A Novel MicroRNA-124/PTPN1 Signal Pathway Mediates Synaptic and Memory Deficits in Alzheimer’s Disease. *Biol Psychiatry* 83 395–405. 28965984 10.1016/j.biopsych.2017.07.023

[B82] Wang Z. Lu G. Sze J. Liu Y. Lin S. Yao H. (2018). Plasma miR-124 Is a Promising Candidate Biomarker for Human Intracerebral Hemorrhage Stroke. *Mol Neurobiol* 55 5879–5888. 29101647 10.1007/s12035-017-0808-8 PMC5994210

[B83] Wu Q. Xu L. Wang C. Fan W. Yan H. Li Q. (2018). MicroRNA-124-3p represses cell growth and cell motility by targeting EphA2 in glioma. *Biochem Biophys Res Commun* 503 2436–2442. 10.1016/j.bbrc.2018.06.173 29969628

[B84] Xu Z. Zhang K. Wang Q. Zheng Y. (2019). MicroRNA-124 improves functional recovery and suppresses Bax-dependent apoptosis in rats following spinal cord injury. *Mol Med Rep* 19 2551–2560. 30720072 10.3892/mmr.2019.9904 PMC6423616

[B85] Yang Y. Ye Y. Kong C. Su X. Zhang X. Bai W. (2019). MiR-124 Enriched Exosomes Promoted the M2 Polarization of Microglia and Enhanced Hippocampus Neurogenesis After Traumatic Brain Injury by Inhibiting TLR4 Pathway. *Neurochem Res* 44 811–828. 30628018 10.1007/s11064-018-02714-z

[B86] Yao G. Li J. Wang J. Liu S. Li X. Cao X. (2020). Improved Resting-State Functional Dynamics in Post-stroke Depressive Patients After Shugan Jieyu Capsule Treatment. *Front Neurosci* 14:297. 10.3389/fnins.2020.00297 32372901 PMC7177051

[B87] Yao L. Ye Y. Mao H. Lu F. He X. Lu G. (2018). MicroRNA-124 regulates the expression of MEKK3 in the inflammatory pathogenesis of Parkinson’s disease. *J Neuroinflammation* 15 13. 29329581 10.1186/s12974-018-1053-4 PMC5767033

[B88] Yao L. Zhu Z. Wu J. Zhang Y. Zhang H. Sun X. (2019). MicroRNA-124 regulates the expression of p62/p38 and promotes autophagy in the inflammatory pathogenesis of Parkinson’s disease. *Faseb j* 33 8648–8665. 30995872 10.1096/fj.201900363R

[B89] Yin H. Kanasty R. L. Eltoukhy A. A. Vegas A. J. Dorkin J. R. Anderson D. G. (2014). Non-viral vectors for gene-based therapy. *Nat Rev Genet* 15 541–555. 10.1038/nrg3763 25022906

[B90] Yu A. Zhang T. Duan H. Pan Y. Zhang X. Yang G. (2017). MiR-124 contributes to M2 polarization of microglia and confers brain inflammatory protection via the C/EBP-α pathway in intracerebral hemorrhage. *Immunol Lett* 182 1–11. 10.1016/j.imlet.2016.12.003 28025043

[B91] Yuan S. Wang Y. X. Gong P. H. Meng C. Y. (2019). MiR-124 inhibits spinal neuronal apoptosis through binding to GCH1. *Eur Rev Med Pharmacol Sci* 23 4564–4574. 31210282 10.26355/eurrev_201906_18032

[B92] Zhang X. Huang X. Fang C. Li Q. Cui J. Sun J. (2017). miR-124 Regulates the Expression of BACE1 in the Hippocampus Under Chronic Cerebral Hypoperfusion. *Mol Neurobiol* 54 2498–2506. 26984601 10.1007/s12035-016-9845-y

[B93] Zhang Y. Liu H. L. An L. J. Li L. Wei M. Ge D. J. (2019). miR-124-3p attenuates neuropathic pain induced by chronic sciatic nerve injury in rats via targeting EZH2. *J Cell Biochem* 120 5747–5755. 10.1002/jcb.27861 30390343

[B94] Zhao Y. Zhang H. Zhang D. Yu C. Y. Zhao X. H. Liu F. F. (2015). Loss of microRNA-124 expression in neurons in the peri-lesion area in mice with spinal cord injury. *Neural Regen Res* 10 1147–1152. 10.4103/1673-5374.156983 26330841 PMC4541249

[B95] Zhou F. Zhang C. Guan Y. Chen Y. Lu Q. Jie L. (2018). Screening the expression characteristics of several miRNAs in G93A-SOD1 transgenic mouse: altered expression of miRNA-124 is associated with astrocyte differentiation by targeting Sox2 and Sox9. *J Neurochem* 145 51–67. 10.1111/jnc.14229 28960306

[B96] Zhou Y. Deng J. Chu X. Zhao Y. Guo Y. (2019). Role of Post-Transcriptional Control of Calpain by miR-124-3p in the Development of Alzheimer’s Disease. *J Alzheimers Dis* 67 571–581. 10.3233/JAD-181053 30584150

